# Influence of dietary linseed oil as substitution of fish oil on whole fish fatty acid composition, lipid metabolism and oxidative status of juvenile Manchurian trout, *Brachymystax lenok*

**DOI:** 10.1038/s41598-019-50243-8

**Published:** 2019-09-25

**Authors:** Jianhua Yu, Shuguo Li, Huaxin Niu, Jie Chang, Zongfu Hu, Ying Han

**Affiliations:** 1 College of Animal Science and Technology, Inner Mongolia University for Nationalities, Tongliao, 028000 China; 2 Department of Animal Science and Technology, Northeast Agricultural University, Harbin, 150030 China

**Keywords:** Ichthyology, Fat metabolism

## Abstract

In this study, juvenile Manchurian trout, *Brachymystax lenok* (initial weight: 6.43 ± 0.02 g, mean ± SE) were received for nine weeks with five types of diets prepared by gradually replacing the proportion of fish oil (FO) with linseed oil (LO) from 0% (LO0) to 25% (LO25), 50% (LO50), 75% (LO75), and 100% (LO100). The eicosapentaenoic (EPA) and docosahexaenoic (DHA) composition decreased with increasing inclusion level of LO (*P* < 0.05). With increasing LO inclusion level, triglyceride (TAG) content of serum increased significantly, however, there was a decrease in high-density lipoprotein cholesterol (HDL) (*P* < 0.05). LO substitution of FO up-regulated the gene expression level of lipid metabolism-related genes Fatty Acid Desaturases 6 (FAD6), Acetyl-Coa Carboxylase (ACCα), Sterol Regulatory Element Binding Protein 1 (SREBP-1), and Sterol O- Acyl Transferase 2 (SOAT2), and down-regulated the gene expression level of Peroxisome Proliferator-Activated Receptor a (PPARα) (*P* < 0.05). The SOD activities of both serum and liver in LO100 were significantly lower than in LO25 (*P* < 0.05). The CAT activity of the liver in LO100 was significantly lower than in LO0 and LO25 (*P* < 0.05). This study indicates that the Manchurian trout may have the ability to synthesize LC-PUFAs from ALA, and an appropriate LO in substitution of FO (<75%) could improve both the lipid metabolism and the oxidation resistance.

## Introduction

Fish oil (FO) is an ideal lipid source in the fish diet since it is rich in highly unsaturated fatty acids (HUFAs), particularly eicosapentaenoic (EPA) and docosahexaenoic (DHA)^1,2^. Both EPA and DHA play important roles in accelerating fish growth and neural development, regulating the fish metabolism, and improving the immunity of the organism^3,4^. However, with the reduction of the global fish meal and fish oil production, finding suitable alternative lipid sources to replace fish oil has become a hot research topic in fish nutrition^5^. Linseed oil (LO) contains an abundance of α-linolenic acid (ALA, C18:3n-3), which is one of the essential fatty acids for freshwater fish. In general, freshwater fish have the ability to synthesize long chain polyunsaturated fatty acids (LC-PUFAs), utilizing ALA; thus, LO has become a potential high-quality substitution oil source^6,7^. Previous studies have shown that FO substitution by a suitable content of LO in the feed would improve the diet conversion rate of fish as well as fish health^6–12^.

However, it has been reported that the absorption of polyunsaturated fatty acids (PUFAs) in fish diet (especially EPA and DHA) will increase the oxidative stress (OS) of the entire organism; furthermore, liver cells might be damaged due to lipid peroxidation, which affects normal physiological function^13^. Addition of a specific proportion of vegetable oil (VO) to the diet reduces the fish’s peroxidation in liver^14^. Therefore, it is of great significance to study the influence of LO substitution of FO on oxidation resistance in fish. Superoxide dismutase (SOD) and catalase (CAT) are the main antioxidant enzymes of the oxidation resistance defense system in fish^15^. Thus, SOD and CAT are common indexes for the oxidation resistance^16,17^.

LO, as a substitution for FO, modulates fish lipid metabolism. The fish’s digestion and absorption of lipids as well as the biological synthesis and decomposition of fatty acids are associated with enzymes related to the fat metabolism and relevant transcription regulators^1^. Fatty acid desaturase 6 (FAD6) controls the synthesis of highly unsaturated fatty acids, and acetyl-CoA carboxylase (ACCα) is the rate-limiting enzyme catalyzing the synthesis of fatty acid^18^. Sterol O-acyl transferase 2 (SOAT2) is mainly involved in the absorption of cholesterol and the assembly of lipoprotein^19^. The sterol regulatory element binding protein-1 (SREBP-1) plays a central role in the control of the fatty acid entry into cholesterol ester^20,21^, and peroxisome proliferators-activated receptors (PPARα) mainly regulate the β-oxidative decomposition of fatty acids^22^.

The Manchurian trout (*Brachymystax lenok*) is a cold-water salmonid fish, which is mainly distributed throughout eastern Siberia, Mongolia, Kazakhstan, Korea, and China. Because it has relatively high nutritious and economic value, it is cultured widely in the northern areas of China^23^. Earlier studies showed that an appropriate lipid level in the diet was favorable for the growth of juvenile Manchurian trout^24^. However, the lipid metabolism and oxidative status of Manchurian trout following LO substitution of FO has not been reported to date. Therefore, the aim of this study was to investigate the influence of FO replacement by LO on both the lipid metabolism and oxidative status of juvenile Manchurian trout by substituting FO with different levels of LO. The appropriate level of LO as FO substitute was comprehensively estimated.

## Materials and Methods

### Ethical standards

All procedures and protocols were approved by the Inner Mongolia University for Nationalities in accordance with national and international guidelines for the care and use of animals in experimentation (2017-IMUN-016).

### Experimental diets

Five groups of diets were prepared with iso-protein (approximately 39.5%) and iso-lipid (approximately 18.5%) using fish meal and soybean meal as the main protein source and FO and LO as the main lipid sources, as shown in Table 1. In each dietary group, the proportion of LO was gradually increased as substitution of FO, with substitution levels of 0% (LO0), 25% (LO25), 50% (LO50), 75% (LO75), and 100% (LO100). Alcohol was used to conduct the degreasing treatment to fish meal used in this experiment to reduce the effects of fish oil in fish meal on the experiment^25^. The raw material of diets was mixed and ground, trace components were mixed and added according to the scale up principle, and finally, the respective lipid was uniformly mixed into the diet. The mixture was extruded as a particle diet (diameter of 1.5 mm) using a DS32-II type two-screw extruder (Jinan Saixin Puffing Machinery Ltd.) after water addition. The mixture was then dried at 50 °C, and stored at −20 °C until future use. Raw materials and rough components are shown in Table 1 and the fatty acid composition of diets is shown in Table 2.

**Table 1:** Formulation and composition of experimental diets.

Ingredient (%)	LO0	LO25	LO50	LO75	LO100
Defatted fishmeal^a^	44	44	44	44	44
Soybean meal	22	22	22	22	22
Gelatin	2	2	2	2	2
Wheat flour	10.45	10.45	10.45	10.45	10.45
Fish oil	16	12	8	4	0
Linseed oil	0	4	8	12	16
Soy lecithin	2	2	2	2	2
Vitamin premix^b^	1.5	1.5	1.5	1.5	1.5
Mineral premix^c^	1.5	1.5	1.5	1.5	1.5
Choline chloride	0.5	0.5	0.5	0.5	0.5
Ethoxy quinoline	0.05	0.05	0.05	0.05	0.05
**Proximate composition (Dry matter)**					
Crude protein	39.45	39.51	39.52	39.49	39.50
Crude lipid	18.36	18.46	18.56	18.43	18.39
Ash	11.29	11.35	11.46	11.29	11.16

^a^Fish meal was defatted with ethanol.

^b^One kilogram of vitamin mix contained the following: VB_1_, 25 mg; VB_2_, 45 mg; VB_6_, 20 mg; VB_12_, 0.l mg; VK_3_, l0 mg; inositol, 800 mg; VB_3_, 60 mg; niacin acid, 200 mg; folic acid, 20 mg; biotin, 1.20 mg; VA, 32 mg; VD_3_, 5 mg; VC, 2150 mg; ethoxyquin, 150 mg; wheat middling, 16.51 g.

^c^One kilogram of mineral mix contained the following: NaF, 200 mg; KI, 80 mg; CoCl_2_·6H_2_O (1%), 5000 mg; CuSO_4_·5H_2_O, 1000 mg; FeSO_4_ · H_2_O, 8000 mg; ZnSO_4_ · H2O, 5000 mg; MnSO_4_·H_2_O, 6000 mg; MgSO_4_ ·7H_2_O, 120000 mg; Ca (H_2_PO_4_)_2_·H_2_O, 750000 mg; NaCl, 1000 mg; zoelite powder, 94270 mg.

**Table 2:** Fatty acid composition (% of total fatty acids) of experimental diets.

Fatty acid	LO0	LO25	LO50	LO75	LO100
C14:0	7.30	5.80	4.30	3.20	1.30
C15:0	1.00	0.80	0.60	0.40	0.12
C18:0	3.80	3.80	3.40	3.10	2.64
C16:0	22.10	18.40	14.90	11.90	8.50
C16:1	6.60	5.30	4.00	2.90	1.80
C18:1	15.40	16.40	17.90	18.20	20.00
C20:1n-9	2.50	3.00	3.20	3.20	3.74
C22:1n-9	3.50	4.50	4.90	5.90	6.60
C18:2n-6	7.10	9.90	12.90	15.80	18.90
C18:3n-3(ALA)	2.20	8.90	16.00	22.90	29.50
C20:3n-3	1.10	0.90	0.70	0.50	0.40
C20:5n-3(EPA)	10.70	8.70	6.70	4.10	2.60
C22:6n-3(DHA)	16.60	13.70	10.60	7.90	3.90
∑SFA	34.20	28.80	23.20	18.60	12.56
∑MUFA	28.00	29.20	30.00	30.20	32.14
∑n-6PUFA	7.10	9.90	12.90	15.80	18.90
∑n-3PUFA	30.60	32.20	34.00	35.40	36.40
DHA + EPA	27.30	22.40	17.30	13.0	6.50

SFA: saturated fatty acids.

MUFA: mono-unsaturated fatty acids.

n-6 PUFA: n-6 poly-unsaturated fatty acids.

n-3 PUFA: n-3 poly-unsaturated fatty acids.

DHA + EPA: C22:6n-3 + C20:5n-3.

### Procedure of the feeding experiment

The Manchurian trout used in this experiment were purchased from the cold-water fish farm of Fengcheng City (Dandong, Liaoning Province, China). The culture experiment was conducted at the Cold Water Fish Culture Laboratory of Inner Mongolia University for Nationalities. Prior to the trial, all Manchurian trout were temporarily cultured for two weeks in an indoor cold-water fish cycling system. During this temporary culture period, Manchurian trout were fed a commercial diet. Prior to the trial, Manchurian trout abstained from eating for 24 hours. Healthy Manchurian trout (average body weight: 6.43 ± 0.02 g, mean ± SE) were selected and randomly stocked into 15 buckets (150 L/bucket, triplicate per diet group), with 20 fish per bucket. Trout were fed until visual satiety twice per day (07:00 and 17:30) with one of the five diets for nine consecutive weeks. A cycling water system was employed with a flow velocity of 1.5 L/min, a water temperature of 15 ± 2 °C, and a dissolved oxygen level >7.5 mg/L.

### Sample collection

At the beginning of the trial, five fish were anaesthetized using MS-222 (100 mg/L), homogenized in a blender and stored at −20 °C for whole-body fatty acid composition analysis. At the end of the trial, fish were anaesthetized, weighted, and counted in each bucket 24 h after feeding. Four fish per bucket were randomly selected and their blood was taken from the tail vein, injected in a centrifuge tube and allowed to clot at 4 °C for 4. Then, the blood was centrifuged for 15 min at 3000 g and 4 °C. The serum was stored in centrifuge tubes and stored at −80 °C for the analysis of blood biochemical index and antioxidant enzyme activity. After taking blood samples, the liver samples (four fish per bucket) were immediately stripped, placed into cryopreservation tubes, and immediately stored in liquid nitrogen at −80 °C for analysis of the metabolism index, oxidative status, and gene expression. In addition, three fish per bucket were selected and homogenized in a blender for the analysis of whole fish crude composition and fatty acid composition. The samples were stored at −20 °C until analysis.

### Proximate composition and fatty acid composition analysis

The AOAC standard method^26^ was adopted for a biochemical composition analysis of both diet and whole fish. All chemical analyses were performed in triplicate. The dry matter was analyzed after drying to a constant weight in a baking oven at 105 °C. The Kjeldahl method (Kjeltec TM 8400, FOSS, Sweden) was adopted to analyze crude protein (total nitrogen × 6.25). The ash content was burnt in a Muffle furnace at 550 °C for 16 h after which, it was measured. The lipids in samples were extracted according to the method of Folch *et al*. (chloroform/mathanol, 2:1,v/v)^27^. Fatty acid methyl esters (FAME) were prepared by the methanol-benzene-acetyl chloride method according to Sukhija and Palmquist^28^. Then, the obtained FAME were analyzed with a Agilent 6890 gas chromatograph (Agilent, USA). The sample (150 mg of dry mass) was added to a hydrolysis tube with 4 ml chloroacetyl methanol solution (chloroacetyl methanol = 1 + 10) and 1 ml C11:0 (1.0 mg/ml) internal standard solution. Then, 1 mL n-hexane was added and reacted in a water bath at 80 °C for 2 h. After cooling, 5 ml of 7% potassium carbonate was added, shaken until an even mixture was obtained, and centrifuged at 1000 r/min for 5 min. Then, the mixture was put into the sample vial through a 0.2 μm filter membrane, and detected by gas chromatograph (Agilent, USA). The chromatographic column was DB-23 (60.0 m × 250 μm × 0.25 μm). He was used as carrier gas and the flow rate was 2.0 ml/min. The shunt ratio was 30:1, the inlet temperature was 260 °C, and the injection volume was 1 ul. The detector was FID and the temperature was 270 °C. The FAs were identified using standard mixtures of methylesters (Supelco 37 component FAME mix), and the FA composition was determined by C11:0.

### Analysis of antioxidant index

The liver samples were homogenized in 10 volumes (w/v) of ice-cold physiological saline and centrifuged at 2500 g for 10 min at 4 °C. Then, the resultant supernatant was conserved until use. Total antioxidant capacity (T-AOC), SOD, and CAT indexes in serum and liver samples were measured based on the relevant toolkits (A015-2-1, A001-3-2 and A007-1-1, Nanjing Jiancheng Bioengineering Ltd., China) according to specification. Corresponding to the 50% inhibition rate of SOD in 1 ml of reaction solution per mL of serum or per mg of histone was defined as one unit of SOD activity. The amount of 1 mol of H_2_O_2_ decomposed per mL of serum or per mg of tissue protein per second was defined as one unit of CAT activity.

### Analysis of blood biochemical index

Serum biochemical index triglyceride (TAG), total cholesterol (TCHO), high density lipid protein cholesterol (HDL-C), and low-density lipid protein cholesterol (LDL-C) were measured according to the relevant toolkits (A110-2-1, A111-2-1, A112-2-1, and A113-2-1, Nanjing Jiancheng Bioengineering Ltd., China) according to specification.

### Real time quantitative PCR

The total RNA in liver was isolated using RNAiso Plus (Takara, Dalian, China), 1.2% agarose gel electrophoresis was used to detect the RNA quality; the RNA concentration was examined using a spectrophotometer at 260 and 280 nm (NanoDrop 2000, Thermo Scientific, USA). According to specifications, 1000 ng RNA was reversed to cDNA via the reverse transcription toolkit (Takara, Dalian, China) following RNA purification. The real time quantitative PCR (RT-qPCR) primers of the lipid metabolism-related gene and the antioxidant gene are shown in Table 3. Expression of the *β-actin* gene is stable and was thus used as RT-qPCR reference gene. To conduct quantitative analysis, the SYBR Premix Ex Taq^TM^II toolkit (Takara, Dalian, China) was used based on the specification of StepOne Plus Real-Time PCR to prepare a standard curve of both the target gene and reference gene as well as to measure the amplification efficiency of both target gene and reference gene. The relative expression levels were normalized to reference gene and calculated using a variation of the Livak and Shmittgen method^29^ corrected for variation in amplification efficiency, as described by Fleige and Pfaffl^30^.

**Table 3:** Primers used in this experiment for RT-qPCR.

Genes	Sense primer	Reversed antisense primer	Size (bp)	Accession no
FAD6	GCATTCCATCCCAATCCTAA	CCCAGGTAGAGGCTGAAGAA	194	MH587164
SOAT2	ACATCGACCAGGGAAGACTG	CCACCGACAGTACCACCTTT	183	MH005798
PPARα	CGGATACCACTACGGAGTTCA	AGGATCTCTGCCTTCAGCTTC	237	MH005796
SREBP-1	CAGGCCTTTAGGGAACACCT	CGGTGACAGTAGCCATGTTG	196	MH005799
ACCa	AGATACGAGGGATGCCACTCT	TCTACCAGGAGGGAACGAGAT	194	MH005800
SOD	CCTTCGGAGACAACACCAAT	AGCCCAGTGAGAGTCAGCAT	181	MH094755
CAT	CGTCAGCCTGTCTACTGCAA	CTTCTCAGCCTGGTCAAAGG	198	MH005801
β-actin	ACTGGGACGACATGGAGAAG	GAGGCGTACAGGGACAACAC	202	KT995162.1

### Statistical analysis

All experimental data are presented as average values ± standard error (means ± SE) and analyzed by one-way analysis of variance (ANOVA) in SPSS19.0. Shapiro-Wilk and Levene tests were used to confirm normality and homogeneity of variance, respectively. Differences between average values were compared using Tukey’s test, and *P* < 0.05 indicates a significant difference.

## Results

### Growth performance and whole-body composition

The growth performance of fish has been reported previously^31^. Briefly, both the final weight (22.33 ± 0.35 g, mean ± SE) and weight gain for the LO100 diet were significantly lower than for the other diets (*P* < 0.05). The feed intake of fish in the LO75 (2.34 ± 0.03%/day, mean ± SE) and the LO100 (2.44 ± 0.02%/day, mean ± SE) diets were significantly higher than for other diets (*P* < 0.05). The feed conversion rate of the LO100 diet was significantly higher than for other diets (*P* < 0.05). LO substitution of FO significantly influenced body composition of Manchurian trout as shown in Table 4. With increasing inclusion level of LO in the diet, the protein content of the fish decreased significantly (*P* < 0.05). The lipid content of the fish in LO25 was highest, and was significantly higher than that in LO50, LO75, and LO100 (*P* < 0.05). No significant differences were found in dry matter and ash contents of fish in all diets (*P* > 0.05).

**Table 4:** Crude composition of the whole-body of Manchurian trout fed with a gradient linseed oil diet.

Proximate composition	LO0	LO25	LO50	LO75	LO100
Dry matter (%)	21.02 ± 0.546^a^	22.66 ± 0.822^a^	22.13 ± 0.484^a^	23.98 ± 0.851^a^	22.39 ± 0.507^a^
Protein(%DM)	58.10 ± 0.091^b^	58.09 ± 0.107^b^	54.59 ± 0.358^a^	54.64 ± 0.636^a^	54.67 ± 0.200^a^
Lipid (% DM)	18.69 ± 0.163^b^	18.90 ± 0.064^b^	17.08 ± 0.187^a^	17.30 ± 0.143^a^	16.67 ± 0.247^a^
Ash (% DM)	9.51 ± 0.193^a^	10.18 ± 0.082^a^	9.88 ± 0.060^a^	9.68 ± 0.279^a^	10.03 ± 0.045^a^

Values are means ± SE from three treatments of fish. Different superscript letters indicate significant differences within the same row (*P* < 0.05).

### Whole fish fatty acid composition

The whole fish fatty acid composition changed in response to the different diets (Table 5). The SFA composition in LO75 and LO100 were significantly higher than in other diets (*P* < 0.05). The MUFA composition increased with increased inclusion level of LO. The n-6 PUFA composition in LO100 was significantly higher than that of other diets except for LO75 (*P* < 0.05). The ALA composition increased with the increase of LO inclusion level (*P* < 0.05). With increased inclusion level of LO in diets, the n-3 LC- PUFA (EPA + DHA) composition decreased significantly (*P* < 0.05).

**Table 5:** Main fatty acid composition (% of total fatty acids) of whole-body Manchurian trout fed with a gradient linseed oil diet.

Fatty acid	Initial	LO0	LO25	LO50	LO75	LO100
C14:0	4.18	4.72 ± 0.118^c^	4.36 ± 0.079^c^	3.55 ± 0.116^b^	2.25 ± 0.052^a^	2.10 ± 0.055^a^
C16:0	25.30	20.11 ± 1.019^c^	19.38 ± 0.870^c^	18.88 ± 0.601^bc^	13.55 ± 0.543^a^	15.80 ± 0.557^ab^
C18:0	7.51	4.42 ± 0.105^a^	4.52 ± 0.116^ab^	4.96 ± 0.151^bc^	4.06 ± 0.081^a^	5.22 ± 0.094^c^
C24:0	0.53	1.56 ± 0.035^d^	1.30 ± 0.038^c^	0.89 ± 0.046^b^	1.04 ± 0.021^b^	0.61 ± 0.009^a^
∑SFA^a^	40.26	33.29 ± 1.277^b^	32.09 ± 1.097^b^	30.56 ± 0.912^b^	22.82 ± 0.696^a^	25.95 ± 0.699^a^
C16:1	4.30	5.30 ± 0.151^c^	4.86 ± 0.090^c^	3.83 ± 0.066^b^	2.41 ± 0.066^a^	2.25 ± 0.066^a^
C18:1n-9c	25.21	16.88 ± 0.592^a^	18.50 ± 0.581^ab^	21.49 ± 1.161^bc^	19.51 ± 0.833^ab^	23.97 ± 1.095^c^
C20:1	1.91	2.14 ± 0.055^a^	2.44 ± 0.035^b^	2.63 ± 0.072^bc^	2.75 ± 0.069^c^	3.17 ± 0.070^d^
C22:1n-9	0.65	0.86 ± 0.038^a^	1.37 ± 0.033^a^	2.22 ± 0.124^b^	2.53 ± 0.048^b^	3.56 ± 0.079^c^
C24:1	1.26	1.19 ± 0.046^a^	1.23 ± 0.035^a^	1.16 ± 0.03^a^	1.05 ± 0.046^a^	1.10 ± 0.041^a^
∑MUFA	33.33	26.37 ± 0.881^a^	28.41 ± 0.750^ab^	31.32 ± 1.234^bc^	28.25 ± 1.056^ab^	33.90 ± 1.206^c^
C18:2n-6c	15.76	10.67 ± 0.612^a^	11.19 ± 0.523^a^	14.34 ± 0.551^b^	16.84 ± 0.623^bc^	17.66 ± 0.615^c^
C20:4n-6	0.53	0.97 ± 0.058^d^	0.82 ± 0.038^c^	0.66 ± 0.009^b^	0.66 ± 0.007^b^	0.50 ± 0.020^a^
∑n-6PUFA^a^	16.62	12.04 ± 0.670^a^	12.33 ± 0.560^a^	15.36 ± 0.560^b^	18.05 ± 0.629^bc^	18.72 ± 0.635^c^
C18:3n-3(ALA)	2.54	4.48 ± 0.118^a^	7.61 ± 0.121^b^	9.55 ± 0.162^c^	14.98 ± 0.578^e^	12.29 ± 0.291^d^
C20:5n-3(EPA)	1.50	6.20 ± 0.173^d^	4.67 ± 0.203^c^	3.18 ± 0.111^b^	3.18 ± 0.061^b^	1.84 ± 0.089^a^
C22:6n-3(DHA)	5.65	17.22 ± 0.575^e^	14.22 ± 0.587^d^	9.69 ± 0.297^b^	12.09 ± 0.069^c^	6.96 ± 0.113^a^
∑n-3PUFA^a^	9.79	28.13 ± 0.863^bc^	26.77 ± 0.904^b^	22.72 ± 0.553^a^	30.75 ± 0.708^c^	21.42 ± 0.483^a^
DHA + EPA	7.15	23.43 ± 0.745^d^	18.89 ± 0.783^c^	12.86 ± 0.392^b^	15.27 ± 0.130^b^	8.80 ± 0.200^a^

Values are means ± SE from three treatments of fish. Different superscript letters indicate significant differences within the same row (*P* < 0.05).

^a^Including some minor components not shown.

### Blood biochemical index

The influence of LO as substitution of FO on serum biochemical index of juvenile Manchurian trout is shown in Table 6. The TAG content significantly increased with increased inclusion level of LO (*P* < 0.05). Compared to LO0, the TCHO content significantly increased in LO25 and LO50 while it decreased in the remaining diets. HDL-C activity showed an initial increase, which was followed by a decrease with increasing LO inclusion level; furthermore, the HDL-C activity in LO25 and LO50 was significantly higher than that in LO0 (*P* < 0.05). LDL-C activity among all groups did not show different changes except LO25, which was significantly lower than for other diets (*P* < 0.05).

**Table 6:** Serum lipid metabolites in Manchurian trout fed with a gradient linseed oil diet.

Parameter	LO0	LO25	LO50	LO75	LO100
TAG (mmol/L)	3.47 ± 0.024^a^	5.39 ± 0.070^b^	9.35 ± 0.102^d^	8.08 ± 0.202^c^	10.37 ± 0.155^c^
TCHO (mmol/L)	7.21 ± 0.034^b^	9.64 ± 0.047^d^	7.93 ± 0.148^c^	6.63 ± 0.199^a^	6.57 ± 0.087^a^
HDL-C (mmol/L)	2.11 ± 0.081^b^	3.23 ± 0.123^d^	2.51 ± 0.091^c^	0.73 ± 0.032^a^	0.79 ± 0.015^a^
LDL-C (mmol/L)	4.38 ± 0.020^b^	3.63 ± 0.139^a^	4.22 ± 0.077^b^	4.12 ± 0.026^b^	4.39 ± 0.091^b^

Values are means ± SE from three treatments of fish (n = 3) with four fish per treatment. Different superscript letters indicate significant differences within the same row (*P* < 0.05).

### Antioxidant index

The influence of LO substitution of FO on antioxidant index of serum and liver of Manchurian trout is shown in Table 7. T-AOC activity of serum in LO25 and LO50 were significantly higher than in other diets (*P* < 0.05). T-AOC activity of the liver gradually decreased, while it was significantly higher than in other diets in LO0 and LO25 (*P* < 0.05). The SOD activities of serum and liver in LO100 were significantly lower than in LO25 (*P* < 0.05). The CAT activity of serum among all different diets were not significantly different (*P* > 0.05). The CAT activity in the liver in LO100 was significantly lower than in LO0 and LO25 (*P* < 0.05).

**Table 7:** Serum and liver antioxidant enzyme activity of Manchurian trout fed with a gradient linseed oil diet.

Parameter	LO0	LO25	LO50	LO75	LO100
**Serum**					
T-AOC(mmol/L)	0.41 ± 0.010^a^	0.58 ± 0.010^b^	0.53 ± 0.021^b^	0.44 ± 0.005 ^a^	0.46 ± 0.007^a^
SOD (units/ml)	127.91 ± 6.087^ab^	136.09 ± 4.069^b^	128.75 ± 2.878^ab^	122.94 ± 2.894^ab^	122.44 ± 1.771^a^
CAT (units/ml)	3.20 ± 0.379^a^	3.52 ± 0.574^a^	3.31 ± 0.208^a^	3.13 ± 0.186^a^	2.82 ± 0.153^a^
**Liver**					
T-AOC (mmol/mg protein)	57.27 ± 0.536^c^	47.63 ± 2.058^b^	41.62 ± 0.949^a^	43.39 ± 1.891^a^	43.18 ± 0.814^a^
SOD (units/mg protein)	440.47 ± 18.369^ab^	459.78 ± 8.778^b^	414.80 ± 13.641^ab^	400.77 ± 14.588^ab^	390.72 ± 12.052^a^
CAT (units/mg protein)	58.01 ± 2.082^bc^	66.33 ± 2.848^c^	47.35 ± 2.400^ab^	47.12 ± 2.312^a^	45.97 ± 1.760^a^

Values are means ± SE from three treatments of fish (n = 3) with four fish per treatment. Different superscript letters indicate significant differences within the same row (*P* < 0.05).

### Expression of lipid metabolism-related genes

**Figure 1:**
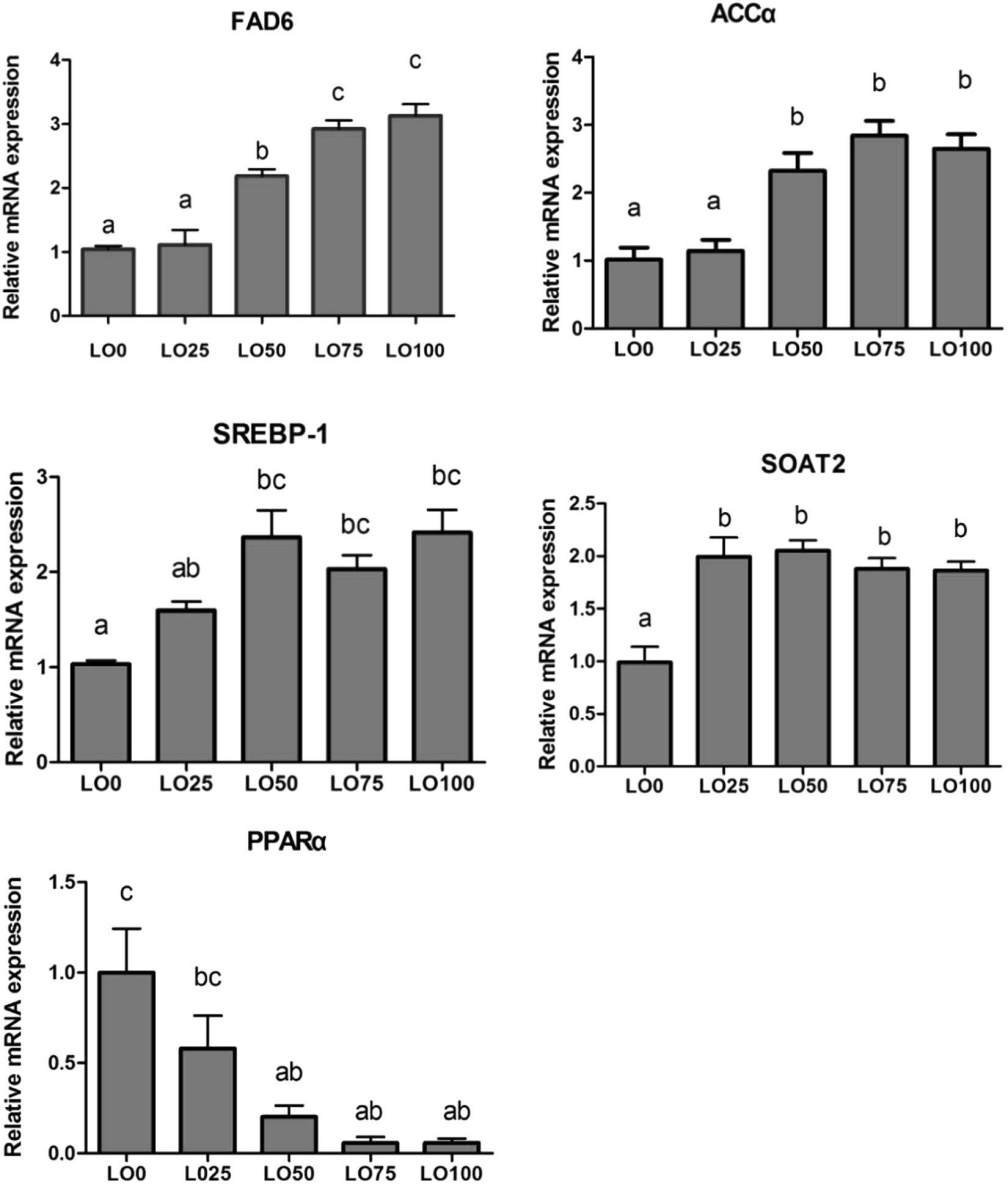
Expression of liver lipid metabolic-related genes (FAD6, ACCα, SREBP-1, SOAT2, and PPARα) in Manchurian trout fed with a gradient linseed oil diet. Values are means ± SE from three treatments of fish (n = 3) with four fish per treatment. Means in each bar that share the different superscript letters indicate significant differences as determined by Tukey’s test (*P* < 0.05).

The influence of LO substitution of FO on the expression of lipid metabolism-related genes of the Manchurian trout is shown in Fig. 1. With increasing LO inclusion level of FO, the expression level of the FAD6 gene gradually increased, and the levels of LO50, LO75, and LO100 were significantly higher than those of other groups (*P* < 0.05). The expression level of the ACCα gene showed an increasing trend with increasing LO inclusion level, and those in LO50, LO75, and LO100 groups were significantly higher than those of the LO0 and LO25 groups (*P* < 0.05). The expression level of the SOAT2 gene in LO0 was significantly lower than that of other diets (*P* < 0.05). The expression level of the SREBP-1 gene in LO0 was significantly lower than that of other diets except LO25 (*P* < 0.05). The expression level of the regulator PPARα gene gradually decreased with increasing LO inclusion level, and levels of LO50, LO75, and LO100 groups were significantly lower than that in LO0 (*P* < 0.05).

### Expression of antioxidant gene

**Figure 2:**
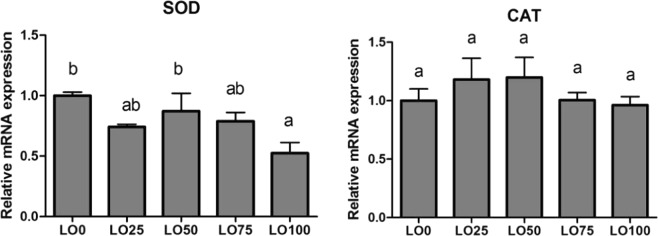
Liver antioxidant gene expressions (SOD and CAT) in Manchurian trout fed with a gradient linseed oil diet. Values are means ± SE from three treatments of fish (n = 3) with four fish per treatment. Means in each bar that share the different superscript letters indicate significant differences as determined by Tukey’s test (*P* < 0.05).

The expression level of the SOD gene of the liver in LO0 was significantly higher than that in LO100 (*P* < 0.05) (Fig. 2). The expression level of the CAT gene was not different among the diets (*P* > 0.05).

## Discussion

ALA has an important physiological function, since it is an essential fatty acid for freshwater fish. LO is one of the best vegetable oil sources with which to substitute FO since LO contains a large amount of ALA^6,7^. In this study, the whole fish protein content gradually decreased after LO replaced FO, where the lipid content was highest at a substitution level of 25% and gradually decreased after that, which was consistent with the results of the Atlantic salmon (*Salmo salar*) and the turbot (*Scophthalmus maximus*)^7,32^. The tissue fatty acid composition of fish was modified by the diets^8^. Similar results were obtained in the present experiment, where the fatty acid composition of whole fish reflected the FA profile of the diet they received. The ALA composition of whole fish increased with the increase of ALA composition in diets. Studies showed that the VO substitution of FO decreased the n-3 LC-PUFA^8,11,33^. In the present experiment, with increased inclusion level of LO in diets (decreased inclusion level of FO), n-3 LC-PUFA (EPA + DHA) decreased significantly.

The LO substitution of FO changed the serum lipid metabolism of Manchurian trout. TAG and TCHO levels reflect the status of an organism’s fat metabolism. In this experiment, the TAG content in serum increased significantly with increasing LO inclusion level (n-3 HUFA content gradually decreased), which was consistent with the results reported by Lemaire *et al*.^34^. In this experiment, with increasing inclusion level of LO, TCHO showed a decreasing trend. Increasing VO levels in aquatic feeds usually reduces dietary cholesterol content and increases phytosterol levels^35^. Similar dietary modifications have been reported to induce a decrease in plasma cholesterol and LDL-C in fish^36–40^. Therefore, lower plasma cholesterol levels in LO rich diets were likely promoted by variations in the dietary cholesterol supply or by the presence of physterol, which interferes with cholesterol absorption. HDL-C played an important role in the regulation of the process of cholesterol transportation from extrahepatic tissues to the liver to be metabolized, and a relatively high level of HDL-C is a feature of wellness of an organism. In this experiment, the HDL-C level in a low substitution treatment (<75%) showed a higher value, indicating that a low LO substitution level (<75%) was favorable for fish metabolism, which was consistent with the results of the turbot^7^.

LO substitution has a specific impact on the synthesis of highly unsaturated fatty acids. FAD6 is the first rate-limiting enzyme of n-3 LC-PUFA synthesis^1^. In this experiment, the expression level of the FAD6 gene increased with increasing LO inclusion level in the diet, which was consistent with the results reported for the silver barb (*Puntius gonionotus*)^11^ as well as the Atlantic salmon^32,41^. LO mainly consists of C18:3n-3, and freshwater fish can convert C18:3n-3 in the diet to HUFA under the effect of FAD6, desaturase, and elongase to compensate for a HUFA deficiency in the diet. Acetyl-CoA carboxylase (ACCα) is the rate-limiting enzyme catalyzing the synthesis of long-chain fatty acids^18^. In this experiment, with increasing LO inclusion level, the expression level of the ACCα gene increased, thus promoting fatty acid synthesis and increasing the TAG content in serum. SOAT2 is the main cholesterol esterase of the liver and the small intestine^19^. LO substitution of FO affected the synthesis of cholesterol of juvenile Manchurian trout. With increasing LO inclusion level, the expression level of the SOAT2 gene increased, which means that the β–oxidation was increased. This was similar to the results for the turbot by Wang *et al*.^7^. SREBP-1 regulates the synthesis of fatty acid and lipid matter^20,21^. The expression level of the SREBP-1 gene showed an increasing trend with increasing inclusion of LO in the diets, which was similar to the results reported by De Tonnac *et al*.^42^ and Li *et al*.^43^. The n-3 HUFA in diet could decrease the expression level of the SREBP-1 gene to down-regulate the gene of synthesizing fatty acid, e.g., the expression of ACCα^18^. PPARα is an essential regulator, regulating both the decomposition and metabolism of fat, and the β-oxidation of fatty acid is mainly realized via PPARα regulation^22^. In this experiment, LO substitution of FO reduced the expression level of the PPARα gene, which was consistent with previous studies^13,43^ suggesting weakened fish β-oxidation ability.

LO substitution of FO changed the fish’s oxidative status. Oxidative stress happens when excess free radicals are generated in the body, such as reactive oxygen species (ROS) and reactive nitrogen species (RNS). When the oxidation degree exceeds the removal of oxides, cells and tissues are damaged^44^. SOD and CAT are among the primary antioxidant enzymes involved in fish antioxidant defense system^15^. SOD is the first enzyme that responds to oxygen radicals, thus preventing the initialization of the chain reaction triggered by superoxide radicals^45^.

In this experiment, SOD activity in serum and liver increased at low LO inclusion levels and decreased in response to high LO inclusion levels. These results showed that the fish’s oxidation resistance did not decrease but rather increased after partial LO substitution of FO, while the oxidation resistance of fish was significantly inhibited after complete substitution. T-AOC is a representation of the overall level of various large and small antioxidant molecules as well as enzymes in a system. In this experiment, T-AOC activity in the serum was highest in LO25 and T-AOC in liver was highest in LO0, indicating that a low LO inclusion proportion was favorable for improving the total oxidation resistance of fish. When the LO inclusion level was too high, the overall oxidation resistance of fish decreased, which has negative effects.

In this experiment, the expression level of SOD and CAT genes in the liver changed in response to increasing LO substitution of FO. CAT removes hydrogen peroxide in the organism by decomposing it into molecule oxygen and water to protect cells from damage by hydrogen peroxide; CAT is therefore, one of the essential enzymes in the biological defense system^46,47^. Although, the expression levels of the CAT gene were not significantly different, the expression levels of the CAT gene at substitution levels of 75% and 100% were lower compared to the LO0 without substitution. These results show that the body was vulnerable to hydrogen peroxide when the level of LO replacement in the diet exceeded 75%. The expression level of SOD in the liver significantly decreased in LO100 compared with other diets in this experiment. The results showed that LO substitution of FO, at high level (100%), decreased the oxidation resistance of fish. In this study, the expression levels of SOD and CAT genes in the liver were basically consistent with the changes of SOD and CAT activities in both serum and liver of fish. This suggested that SOD and CAT expression levels in the liver of fish might be correlated with SOD and CAT activities in both serum and liver of fish. T-AOC activity was highest in LO0 diet (100% FO) in liver, but in serum, it was higher for LO25 and LO50 diets. The mechanism of tissue difference of T-AOC activity between serum and liver requires further study.

In summary, the Manchurian trout (*Brachymystax lenok*) may have the ability to synthesize LC-PUFAs from ALA. LO substitution of FO at an appropriate proportion in the diet (<75%) improved both lipid metabolism and the oxidative status of juvenile Manchurian trout.
